# Association Between Red Blood Cell Distribution Width and Thyroid Function

**DOI:** 10.3389/fendo.2021.807482

**Published:** 2022-01-18

**Authors:** Guowei Zhou, Yueqin Ai, Song Guo, Quan Chen, Xiao Feng, Kun Xu, Gaoyuan Wang, Chaoqun Ma

**Affiliations:** ^1^ Department of General Surgery, Jiangsu Province Hospital of Chinese Medicine, Affiliated Hospital of Nanjing University of Chinese Medicine, Nanjing, China; ^2^ Department of Pneumology, Jinling Hospital, Nanjing, China

**Keywords:** red blood cell distribution width, thyroid, subclinical hypothyroidism, NHANES, cross-sectional study

## Abstract

**Aim:**

Red blood cell distribution width (RDW) is an important parameter with broad biological implications. However, the study investigating the association between RDW and thyroid function remains sparse and inconsistent. We aimed to investigate the association between RDW and thyroid function in the US population.

**Methods:**

A cross-sectional analysis was performed using the data from the National Health and Nutrition Examination Survey (NHANES) conducted from 2007 to 2010. The thyroid parameters investigated were mainly free triiodothyronine (fT3), free thyroxine (fT4), thyroid-stimulating hormone (TSH), antithyroglobulin antibody (TgAb), and antithyroperoxidase antibody (TPOAb). In the 6,895 adults aged 18 years or older, logistic regression modeling was applied to estimate the association between RDW quartiles and thyroid parameters. Smooth curve fittings and generalized additive models were then performed to address the nonlinear relationship.

**Results:**

The association between RDW and TSH followed a J-shaped curve, and a significant positive relationship existed in the 12.5%–17.5% range of RDW (*β*  = 0.350, 95% confidence interval (CI): 0.225 to 0.474), which was prominent in females. We further demonstrated a negative association (*β* = −0.018, 95% CI: −0.030 to −0.005) between RDW and fT3. Moreover, elevated RDW was more likely to be subclinical hypothyroidism. However, there was no obvious association between RDW and fT4.

**Conclusion:**

This study confirmed a significant association between RDW and TSH, and future studies are needed to elucidate the underlying mechanisms of the peculiar RDW-fT3 relationship. RDW may be a significant clinical marker of subclinical hypothyroidism.

## Introduction

Red blood cell distribution of width (RDW), a component of the standard complete blood count (CBC), is an indicator of the heterogeneity of erythrocyte volume (anisocytosis), and elevated RDW implicates homeostatic imbalance of erythrocyte. RDW was traditionally regarded as a part of routine evaluation of anemia (1, 2), especially iron-deficiency anemia (3). Nonetheless, the clinical application of RDW has been considered far beyond the differential diagnosis of anemias, and there have been substantial researches undertaken on the role of RDW in acute or chronic human disorders characterized by inflammation (4), which further attested that RDW is an independent risk factor for death in the general population with or without common comorbidities, such as cardiovascular diseases (CVD) (5–7), chronic obstructive pulmonary disease (COPD) (8), cancer (9, 10), celiac disease (11), and other conditions (1, 12).

Many studies have revealed the tight linkage between anemia and thyroid dysfunction (3, 13, 14). Extensive researches have implied that thyroid hormones (THs) played a significant role in regulating hematopoiesis by increasing renal erythropoietin (EPO) mRNA expression and EPO production (13), stimulating bone marrow erythropoiesis (15–17), and regulating the proliferation of erythroid precursors (15, 17). However, the study investigating the association between RDW and thyroid status remains sparse and inconsistent. Data from several studies have indicated that elevated RDW level was significantly associated with Hashimoto thyroiditis (HT) (18), subclinical hypothyroidism (19), hyperthyroidism, and hypothyroidis (20). However, in a study by Lippi et al. (21), RDW was not correlated with thyroid-stimulating hormone (TSH) in the euthyroid older patients. Moreover, the nonsignificant association between RDW and thyroid function was also observed in the Chinese population studied by Wang et al., which further suggested that anisocytosis might be a contributing factor in thyroid dysfunction (22).

Hence, for providing more evidence to resolve the contradiction mentioned above, the current thesis intends to investigate the cross-sectional association between RDW and thyroid function in a nationally representative sample of the civilian noninstitutionalized adult population in the United States (US) from the National Health and Nutrition Examination Survey (NHANES) from 2007 to 2010.

## Materials and Methods

### Study Population

The NHANES is a biennial program conducted by the Centers for Disease Control and Prevention to assess the nutrition and health status of the US population. The survey utilizes a complex multistage cluster design and includes extensive demographics, clinical interviews, physical examinations, and laboratory components. Prior to data collection, the study procedures of NHANES have been approved by the Institutional Review Board (IRB) of the National Center for Health Statistics (NCHS), with written informed consent obtained.

**Figure 1:**
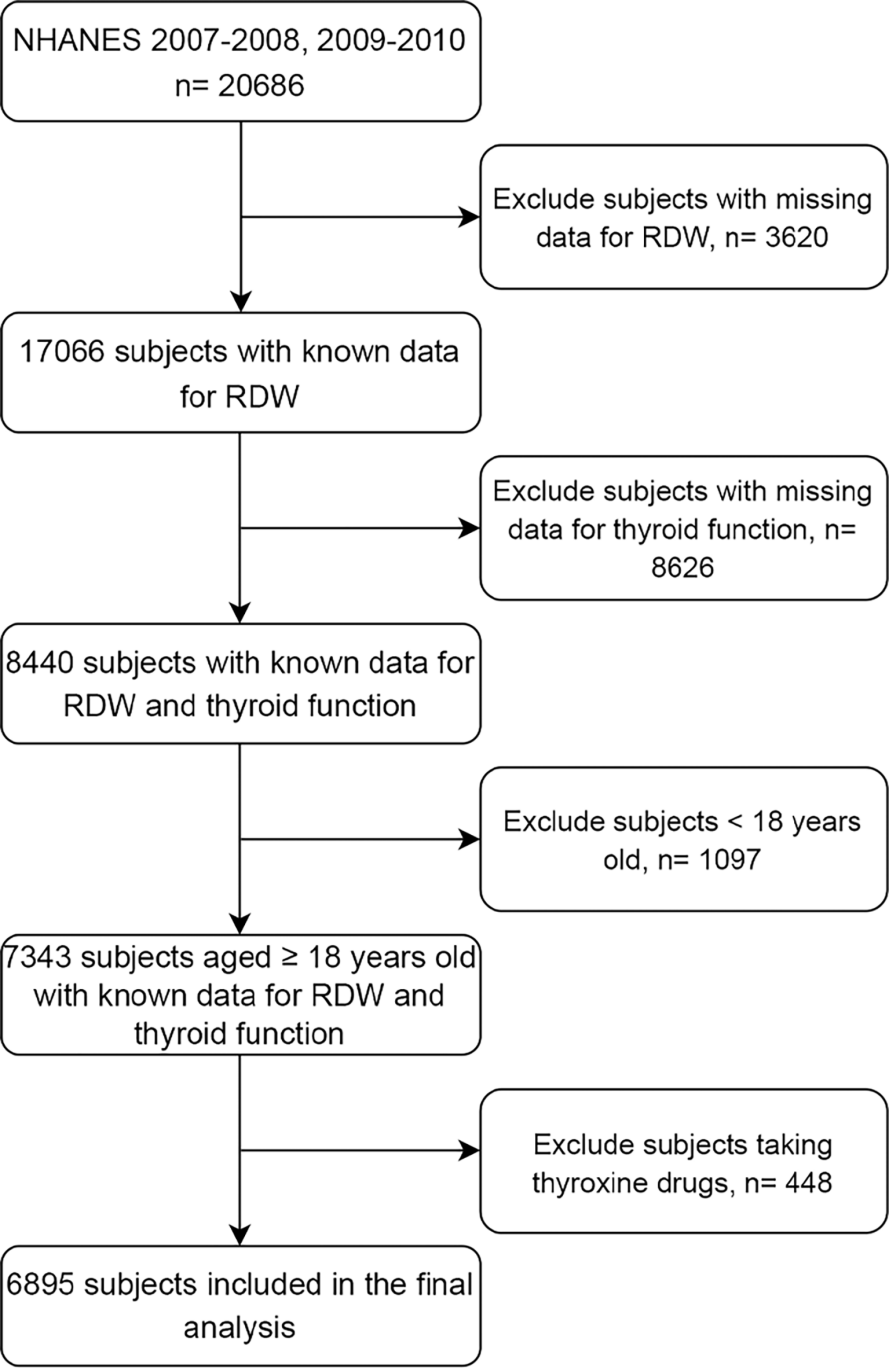
Study flowchart. NHANES, National Health and Nutrition Examination Survey; RDW, red blood cell distribution width.

In the present study, we merged two waves of NHANES: NHANES 2007–2008 (“E” data) and NHANES 2009–2010 (“F” data). The variables investigated in this work were obtained following protocols available in NHANES operation manuals, which were discussed on the NHANES website. The participants aged 18 years or older and had RDW, as well as thyroid function laboratory data, were included (**Figure 1**).

### Measurement of RDW

The RDW (%) was measured by the Coulter analyzer in the Mobile Examination Centers (MECs), of which the normal range was 11.5%–14.5% (23, 24). In the current study, we investigated RDW as a continuous and categorical variable in quartile (Q1 = 10.8%–12.1%, Q2 = 12.2%–12.5%, Q3 = 12.6%–13.2%, Q4 = 13.3%–37.8%).

### Thyroid Outcomes

The thyroid parameters investigated in this study included free triiodothyronine (fT3), free thyroxine (fT4), TSH, total T3 (TT3), total T4 (TT4), thyroglobulin (Tg), antithyroglobulin antibody (TgAb), and antithyroperoxidase antibody (TPOAb), and detailed specimen collection and processing instructions are available in the NHANES Laboratory/Medical Technologists Procedures Manual (LPM).

Briefly, the TSH level was detected by a third-generation, two-site immunoenzymatic (“sandwich”) assay (reference range, 0.34–5.6 µIU/ml). The fT3 assay was a competitive binding immunoenzymatic assay (reference range, 2.5–3.9 pg/ml) and the fT4 assay was a two-step enzyme immunoassay (reference range, 0.6–1.6 ng/dl). In the present work, subjects with the levels of TSH and fT4 within the reference range were categorized as euthyroid (EU). We then defined hypothyroidism as TSH ≥5.6 µIU/ml, either subclinical (SHypo), with normal fT4 or overt (OHypo) in combination with reduced fT4 concentration. Similarly, hyperthyroidism was defined as TSH ≤0.44 mIU/L, either subclinical (SHyper) in combination with normal fT4, or overt (OHyper) with elevated fT4 (25, 26).

Both TPOAb and TgAb titers were measured with a sequential two-step immunoenzymatic (“sandwich”) assay, with reference ranges of 0–9.0 IU/ml and 0–4.0 IU/ml, respectively. Thyroid autoimmunity was defined as TPOAb titers >9.0 IU/ml and/or TgAb titers >4.0 IU/ml (27). In addition, the NHANES survey records a list of prescription medications, including levothyroxine (drug code, d00278), liothyronine (h00019), methimazole (d00290), and propylthiouracil (d00361). To decrease the effect of the thyroid gland disease-related medications on thyroid function and thyroid autoimmunity, we decided to exclude the subjects taking the thyroxine drugs mentioned above (**Figure 1**).

### Demographic and Social Characteristics

Information regarding age, gender, race/ethnicity, education, marital status, poverty-to-income ratio (PIR), medical history (including congestive heart failure, coronary heart disease, angina pectoris, and cancer), tobacco use, and alcohol consumption was acquired using a standardized questionnaire, and the values of mean arterial pressure (MAP) and body mass index (BMI) were collected in the MECs by well-trained health technicians.

Race/ethnicity was coded into four groups as Non-Hispanic White, Non-Hispanic Black, Mexican American, and Other Hispanic, and education level was categorized as less than high school diploma, high school diploma, and more than high school diploma. PIR was employed to evaluate the socioeconomic status (SES) of the participants, which was categorized as 0–1 and >1. Smoking status was coded as never, former, and current. Never smokers were the participants reported smoking less than 100 cigarettes during their life, while former smoking was defined as having smoked more than 100 cigarettes during the lifetime, but not currently smokers. Similarly, alcohol use was categorized into four spices from never up to four or more drinks per day for men, and three or more for women, respectively (28). BMI was calculated as weight in kilograms divided by height in square meters, and the formula for MAP was: [(diastolic blood pressure × 2) + systolic blood pressure]/3.

### Laboratory Procedures

The laboratory indices researched in this work included hemoglobin (g/dl), mean corpuscular volume (MCV, fl), total cholesterol (mg/dl), HDL cholesterol (mg/dl), triglycerides (TC, mg/dl), serum albumin (g/L), blood urea nitrogen (BUN, mg/dl), serum iron (µg/dl), erythrocyte folate (nmol/L), serum cotinine (ng/ml), C-reactive protein (CRP, mg/dl), urine iodine (µg/L), and creatinine (µmol/L), which were recorded by certified technicians using standardized laboratory methods. To assess renal function, the estimated glomerular filtration rate (eGFR) was calculated using the following modification of diet in renal disease equation: eGFR = 175 * (standardized creatinine/88.4)^−1.154^ * (age)^−0.203^ * (0.742 if the participant is female) * (1.212 if the participant is black) × 0.0167 (2, 29). In addition, urine iodine was categorized into three groups as <99, 99–199, and >199 µg/L (27).

### Statistical Analysis

Sampling weights were calculated, as recommended by NHANES, to adjust for the complex sample design and produce corrected estimates of standard errors (30). Since all of the continuous variables included in the present study did not subject to the normal distribution, we performed Kruskal-Wallis (KW) test for the comparison of continuous variables among RDW groups, which were then described in terms of median and 95% confidence limit estimated from weighted frequencies. Whereas categorical variables were summarized using frequency counts and weighted percentages.

Weighted multivariable linear and logistic regression analyses were employed to investigate the association between RDW with thyroid parameters. For the linear regression models, RDW was treated as a continuous variable, while for the logistic models, RDW was categorized into quartile, and then, we entered the median value of each category of RDW as a continuous variable for the tests of linear trend (31). Furthermore, interaction and stratified analyses were conducted according to gender. In addition, for addressing the nonlinear relationship, smooth curve fittings and generalized additive models were applied in the current study, and the inflection points were further calculated using a recursive algorithm.

The statistical software packages R 4.1.0 (http://www.R-project.org) and EmpowerStats (http://www.empowerstats.com, X&Y Solutions, Inc., Boston, MA) were applied for statistical analyses, with a *p* value <0.05 considered statistically significant.

## Results

### Subjects and Demographic Characteristics

A total of 6895 participants were included in the current study from the NHANES 2007-2010 (**Figure 1**). Baseline participant characteristics, stratified according to RDW quartiles, were shown in **Supplementary Table S1**. The participants with elevated RDW levels were more likely to be older, female, less educated, obese, and with lower SES and higher MAP. The weighted percentage of non-Hispanic blacks and former smoking, as well as the levels of HDL cholesterol, CRP, and serum cotinine, increased with increasing RDW quartiles, while MCV, serum albumin, serum iron, and estimated GFR levels decreased with elevated RDW levels. In addition, despite the significant differences in alcohol use, urine iodine, as well as HB, TC, and BUN levels between RDW quartiles, the relationship did not follow a linear trend.

As shown in **Table 1**, the participants included in the current study were mostly with normal thyroid function (*n* = 6,296, 92.9%), and the numbers for the OHyper, SHyper, SHypo, and OHypo were respectively 194 (2.9%), 12 (0.2%), 243 (3.6%), and 28 (0.4%). Meanwhile, the majority of the 6,895 participants (*n* = 6,081, 88.2%) were present without thyroid autoimmunity. Furthermore, a significant linear negative correlation existed for fT3 and TT3 levels between RDW quartiles, while the positive correlation for Tg levels. Elevated RDW levels were more likely to be subclinical hypothyroidism. However, the correlation in the levels of TSH and TT4 between RDW quartiles did not follow a linear relationship, furthermore, the correlation in fT4 and thyroid autoimmunity was not significantly different.

**Table 1:** Thyroid function of NHANES (2007–2010) study population in RDW quartiles.

Characteristics	RDW quartiles					*p*-value
	Overall	Q1	Q2	Q3	Q4	
*n*	6,895	1,593	1,678	1,879	1,745	
RDW (%)	12.6 (10.8–37.8)	10.8–12.1	12.2–12.5	12.6–13.2	13.3–37.8	
**Thyroid function**						
FT3 (pg/ml)	3.20 [2.90–3.40]^a^	3.22 [3.00–3.50]	3.20 [3.00–3.50]	3.10 [2.90–3.40]	3.08 [2.80–3.30]	<0.001
FT4 (ng/dl)	0.80 [0.70–0.88]	0.80 [0.70–0.86]	0.80 [0.70–0.84]	0.80 [0.70–0.88]	0.80 [0.70–0.90]	0.748
Tg (ng/ml)	10.15 [5.92–17.63]	9.33 [5.68–14.77]	9.60 [5.79–16.55]	10.70 [5.99–18.42]	11.33 [6.29–20.34]	<0.001
TSH (µIU/ml)	1.55 [1.04–2.32]	1.51 [1.04–2.18]	1.53 [1.01–2.33]	1.59 [1.06–2.38]	1.57 [1.03–2.39]	0.030
TT3 (ng/dl)	112.00 [99.00–127.00]	115.00 [102.00–130.00]	114.50 [102.00–129.00]	112.00 [98.00–126.00]	107.00 [94.00–122.00]	<0.001
TT4 (ng/dl)	7.70 [6.80–8.70]	7.70 [6.80–8.70]	7.70 [6.80–8.60]	7.60 [6.80–8.70]	7.80 [6.80–8.90]	0.040
Categories						
OHyper	194 (2.9%)^b^	39 (2.5%)	55 (3.3%)	47 (2.6%)	53 (3.1%)	0.016
SHyper	12 (0.2%)	2 (0.1%)	3 (0.2%)	3 (0.2%)	4 (0.2%)
EU	6269 (92.9%)	1484 (94.3%)	1536 (92.9%)	1709 (93.2%)	1540 (91.3%)
SHypo	243 (3.6%)	44 (2.8%)	55 (3.3%)	69 (3.8%)	75 (4.4%)
OHypo	28 (0.4%)	5 (0.3%)	4 (0.2%)	5 (0.3%)	14 (0.8%)
**Thyroid autoimmunity**						
TgAb (IU/ml) <4 (%)						
Yes	6,503 (94.3%)	1,504 (94.4%)	1,592 (94.9%)	1,771 (94.3%)	1,636 (93.8%)	0.415
No	392 (5.7%)	89 (5.6%)	86 (5.1%)	108 (5.7%)	109 (6.2%)
TPOAb (IU/ml) <9 (%)						
Yes	6,270 (90.9%)	1,451 (91.1%)	1,532 (91.3%)	1,713 (91.2%)	1,574 (90.2%)	0.186
No	625 (9.1%)	142 (8.9%)	146 (8.7%)	166 (8.8%)	171 (9.8%)
Thyroid autoimmunity (%)						
Yes	814 (11.8%)	181 (11.4%)	188 (11.2%)	221 (11.8%)	224 (12.8%)	0.193
No	6,081 (88.2%)	1,412 (88.6%)	1,490 (88.8%)	1,658 (88.2%)	1,521 (87.2%)

a Weighted median [95% CI for median].

b Actual frequency (weighted percentage).

NHANES, National Health and Nutrition Examination Survey; RDW, red blood cell distribution width; fT3, free triiodothyronine; fT4, free thyroxine; TSH, thyroid-stimulating hormone; TT3, total T3; TT4, total T4; Tg, thyroglobulin; EU, euthyroid; SHypo, subclinical hypothyroidism; OHypo, overt hypothyroidism; SHyper, subclinical hyperthyroidism; OHyper, overt hyperthyroidism; TgAb, antithyroglobulin antibody; TPOAb, antithyroperoxidase antibody.

### Association Between RDW and TSH

**Figure 2:**
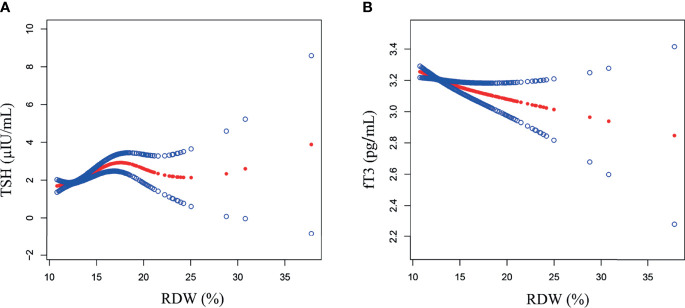
The association between RDW and TSH, as well as fT3. Solid red line represents the smooth curve fit between RDW and TSH **(A)**, as well as fT3 **(B)**. Blue bands represent the 95% of confidence interval from the fit. Age, gender, race/ethnicity, education, poverty-to-income ratio, mean arterial pressure, body mass index, alcohol use, smoke, hemoglobin, mean corpusular volume, total cholesterol, high-density lipoprotein cholesterol, serum albumin, serum iron, blood urea nitrogen, serum cotinine, estimated glomerular filtration rate, C-reactive protein, and urine iodine were adjusted. TSH, thyroid-stimulating hormone; fT3, free triiodothyronine; RDW, red blood cell distribution width.

As demonstrated on **Table 2**, RDW was positively correlated with TSH (*β* = 0.095, 95% CI: 0.036 to 0.153, *p* = 0.002) in the unadjusted model, and the positive association was still present in model 2 (*β* = 0.109, 95% CI: 0.044 to 0.173, *p* = 0.002) and model 3 (*β* = 0.132, 95% CI: 0.056 to 0.208, *p* < 0.001) after adjusting the confounders. After categorizing RDW into quartiles, participants in the highest RDW quartile had a 0.237-µIU/ml greater TSH than those in the lowest RDW quartile (*p* = 0.046). In further analysis, the association between RDW and TSH followed a J-shaped curve (**Figure 2A**), and then, the two-piecewise linear regression model was applied to obtain the inflection points. As shown in **Table 3**, a significant positive relationship existed for TSH and RDW range from 12.5% to 17.5% (*β* = 0.350, 95% CI: 0.225 to 0.474, *p* < 0.001), while the correlation was not significant when RDW level <12.5% (*β* = −0.119, 95% CI: −0.366 to 0.129, *p* = 0.348) and >17.5% (*β* = 0.043, 95% CI: −0.197 to 0.283, *p* = 0.726). In addition, in the subgroup analysis stratified by sex, as adjusting the confounders, the positive relationship between RDW and TSH still existed in female subjects (*β* = 0.192, 95% CI: 0.060 to 0.323, *p* = 0.004), however was not present in male participants (*β* = 0.034, 95% CI: −0.024 to 0.093, *p* = 0.252). Nonetheless, the further interaction analysis indicated that sex was not the interactive factor in the correlation between RDW and TSH.

**Table 2:** The association between RDW (%) and TSH (µIU/ml).

	Model 1^a^	Model 2^b^	Model 3^c^
	*β* (95% CI), *p*-value	*β* (95% CI), *p*-value	*β* (95% CI), *p*-value
**Total**			
RDW (%)	0.095 (0.036, 0.153), 0.002	0.109 (0.044, 0.173), 0.002	0.132 (0.056, 0.208), <0.001
RDW categories			
Q1 (10.8%–12.1%)	Reference	Reference	Reference
Q2 (12.2%–12.5%)	−0.087 (−0.275, 0.101), 0.365	−0.0101 (−0.298, 0.095), 0.352	−0.071 (−0.269, 0.127), 0.480
Q3 (12.6%–13.2%)	−0.061 (−0.249, 0.128), 0.529	−0.124 (−0.326, 0.079), 0.233	−0.101 (−0.307, 0.105), 0.336
Q4 (13.3%–37.8%)	0.202 (−0.001, 0.405), 0.051	0.217 (−0.009, 0.444), 0.093	0.237 (0.002, 0.487), 0.046
* p* for trend	0.105 (0.007, 0.202), 0.036	0.095 (−0.016, 0.205), 0.093	0.112 (−0.007, 0.224), 0.065
**Subgroup analysis stratified by sex**			
** Sex = male**			
RDW (%)	0.041 (−0.007, 0.089), 0.095	0.018 (−0.035, 0.072), 0.504	0.034 (−0.024, 0.093), 0.252
** Sex = female**			
RDW (%)	0.108 (0.012, 0.204), 0.028	0.141 (0.037, 0.246), 0.008	0.192 (0.060, 0.323), 0.004
* p* for interaction	0.302	0.148	0.206

a Model 1: no covariates were adjusted.

b Model 2: age, gender, and race/ethnicity were adjusted.

c Model 3: age, gender, race/ethnicity, education, poverty-to-income ratio, mean arterial pressure, body mass index, alcohol use, smoke, cancer, coronary heart disease, congestive heart failure, angina pectoris, hemoglobin, mean corpusular volume, total cholesterol, high-density lipoprotein cholesterol, serum albumin, serum iron, blood urea nitrogen, serum cotinine, estimated glomerular filtration rate, C-reactive protein, and urine iodine were adjusted. In the subgroup analysis stratified by sex, the model was not adjusted for sex.

RDW, red blood cell distribution width; TSH, thyroid-stimulating hormone; CI, confidence interval.

**Table 3:** Threshold effect analysis of RDW on TSH using the two-piecewise linear regression model.

TSH	Adjusted *β* (95% CI), *p*-value
**Fitting by the standard linear model**	0.132 (0.056, 0.208) <0.001
**Fitting by the two-piecewise linear model**	
Inflection points	12.5, 17.5
RDW <12.5%	−0.119 (−0.366, 0.129), 0.348
12.5% ≤RDW ≤17.5%	0.350 (0.225, 0.474), <0.001
RDW >17.5%	0.043 (−0.197, 0.283), 0.726
Log likelihood ratio	<0.001

Age, gender, race/ethnicity, education, poverty-to-income ratio, mean arterial pressure, body mass index, alcohol use, smoke, cancer, coronary heart disease, congestive heart failure, angina pectoris, hemoglobin, mean corpusular volume, total cholesterol, high-density lipoprotein cholesterol, serum albumin, serum iron, blood urea nitrogen, serum cotinine, estimated glomerular filtration rate, C-reactive protein, and urine iodine were adjusted.

RDW, red blood cell distribution width; TSH, thyroid-stimulating hormone; CI, confidence interval.

### Association Between RDW and fT3

**Table 4** and **Figure 2B** indicate a negative linear association between RDW and fT3 in the unadjusted model (*β* = −0.039, 95% CI: −0.050 to −0.029, *p* < 0.001), model 2 (*β* = −0.024, 95% CI: −0.035 to −0.013, *p* < 0.001) and model 3 (*β* = −0.018, 95% CI: −0.030 to −0.005, *p* = 0.006), which was still present when RDW coded into quartiles (*p* for trend <0.001). Furthermore, the negative association remained in both female (*β* = −0.024, 95% CI: −0.044 to −0.004, *p* = 0.022) and male (*β* = −0.014, 95% CI: −0.026 to −0.003, *p* = 0.001) subjects in the subgroup analysis, and the interaction analysis revealed that gender did not play an interactive role in the relationship between RDW and fT3 (*p* = 0.945).

**Table 4:** The association between RDW (%) and fT3 (pg/ml).

	Model 1^a^	Model 2^b^	Model 3^c^
	*β* (95% CI), *p*-value	*β* (95% CI), *p*-value	*β* (95% CI), *p*-value
**Total**			
RDW (%)	−0.039 (−0.050, −0.029), <0.001	−0.024 (−0.035, −0.013), <0.001	−0.018 (−0.030, −0.005), 0.006
RDW categories			
Q1 (10.8%–12.1%)	Reference	Reference	Reference
Q2 (12.2%–12.5%)	−0.031 (−0.064, 0.002), 0.063	−0.009 (−0.042, 0.024), 0.600	−0.015 (−0.048, 0.018), 0.378
Q3 (12.6%–13.2%)	−0.093 (−0.126, −0.060), <0.001	−0.048 (−0.082, −0.014), 0.006	−0.042 (−0.076, −0.008), 0.016
Q4 (13.3%–37.8%)	−0.151 (−0.187, −0.116), <0.001	−0.094 (−0.132, −0.056), <0.001	−0.026 (−0.038, −0.013), <0.001
* p* for trend	−0.06 (−0.07, −0.05), <0.001	−0.04 (−0.05, −0.02), <0.001	−0.026 (−0.038, −0.013), <0.001
**Subgroup analysis stratified by sex**			
** Sex = male**			
RDW (%)	−0.082 (−0.096, −0.069), <0.001	−0.024 (−0.037, −0.010), <0.001	−0.014 (−0.026, −0.003), 0.001
** Sex = female**			
RDW (%)	−0.020 (−0.035, −0.005), 0.011	−0.023 (−0.039, −0.007), 0.005	−0.024 (−0.044, −0.004), 0.022
* p* for interaction	0.068	0.346	0.945

a Model 1: no covariates were adjusted.

b Model 2: age, gender, and race/ethnicity were adjusted.

c Model 3: age, gender, race/ethnicity, education, poverty-to-income ratio, mean arterial pressure, body mass index, alcohol use, smoke, cancer, coronary heart disease, congestive heart failure, angina pectoris, hemoglobin, mean corpusular volume, total cholesterol, high-density lipoprotein cholesterol, serum albumin, serum iron, blood urea nitrogen, serum cotinine, estimated glomerular filtration rate, C-reactive protein, and urine iodine were adjusted. In the subgroup analysis stratified by sex, the model was not adjusted for sex.

RDW, red blood cell distribution width; fT3, free triiodothyronine; CI, confidence interval.

## Discussion

In this cross-sectional investigation, we used the NHANES 2007–2010 database to demonstrate a relationship between RDW and thyroid parameters in a nationally representative cohort in the US population. The present study indicated that the association between RDW and TSH followed a J-shaped curve, and a significant positive relationship existed in the 12.5%–17.5% range of RDW, which was more prominent in females. In addition, elevated RDW level was significantly associated with decreasing fT3 level and more likely to be subclinical hypothyroidism. However, there was no obvious association between RDW and fT4, as well as thyroid autoimmunity.

RDW is a simple and inexpensive hematological parameter reflecting the anisocytosis and is clinically applied as the laboratory marker in the differential diagnosis of anemias (1). Substantial researches have implied that elevated RDW may mirror the dysfunction of iron metabolism and increased systemic inflammation (32–36). Anemia and thyroid dysfunction are common disorders and often occur simultaneously (37, 38). Prior investigations have revealed a significant association between thyroid function and erythrocyte indices, including Hb, hematocrit, and erythrocyte count (39, 40). Gu et al. demonstrated the function of low-normal THs in predicting future anemia and annual changes in Hb in the general population (16). Furthermore, thorough studies revealed the multiple mechanisms of THs in stimulating erythropoiesis, including affecting iron transport and utilization, as well as stimulating erythropoietin production and responsiveness (39, 41–44). The works mentioned above suggested the underlying association between RDW and thyroid status; however, the current studies regarding the relationship between RDW and thyroid function were sparse and inconsistent.

TSH is a glycoprotein hormone secreted by the pituitary, which plays a crucial role in stimulating thyroid growth and differentiation, as well as regulating the synthesis and secretion of THs (45). In a report from Italy involving 8,477 Caucasian participants, Montagnana et al. demonstrated a significant positive relationship between TSH (mIU/L) and RDW (*β* = 0.079, *p* < 0.001) in the multiple linear regression analysis (20). In the same vein, Yu et al. also revealed the independent, significant association between TSH levels (mU/L) and RDW (*β* = 0.102, *p* < 0.001) in a large healthy population (19). The findings of the prior studies supported the results of the present study. In addition, as shown in **Figure 2A** and **Table 3**, we further found a J-shaped pattern between RDW and TSH, as well as a significant positive correlation in the 12.5%–17.5% range of RDW. However, in a study involving 1,050 euthyroid participants aged 50 years or older, Lippi et al. indicated that, although the values of RDW was gradually increasing from the first to the fourth quartile of TSH, none of the hematological parameters were correlated with TSH in the multivariable linear regression analysis, RDW included (*p* = 0.24) (21). Nonetheless, Lippi et al. confirmed the pivotal role of thyroid status in anisocytosis (21). Moreover, in the current work, although the interaction analysis exhibited that gender was not the interactive factor, the significant positive correlation between RDW and TSH appeared to be more prominent in females, and the sex difference has also been reported in the investigations by Lippi et al. (21) and Wang et al. (22). Hence, we suggested that more attention should be paid to the relevance between thyroid dysfunction and anisocytosis in the female subjects.

As shown in **Table 1**, SHypo was the most common thyroid dysfunction in our population. Similar to the study by Yu et al. (19), our study also revealed the significant relationship between increasing RDW and SHypo. Interestingly, although the present study exhibited a significant negative relevance between RDW and fT3, there was no obvious association between RDW and fT4. Similar phenomenon was reported in the previous studies (46, 47). In a study by pulsed wave tissue Doppler imaging (PWTDI), Zoncu et al. attested that PWTDI parameters were more likely to be correlated with fT3 concentrations, but not with fT4, which further proved the crucial role of T3 in heart function, and the compromised systolic function in SHypo patients (47). The peculiar relationship was also reported in the study by Ruscica et al., which revealed that leptin was significantly correlated with fT3 rather than fT4 levels (46). Illuminatingly, the animal experiments implied the underlying mechanism that leptin may regulate the activity of hypothalamic, pituitary, and peripheral 5′ deiodinase, and then reduce the conversion of T4 to T3 (48). Overall, although there is as yet no definite explanation of the mechanism underlying the peculiar RDW-fT3 relationship, it seemed logical when considering the critical roles both of RDW and fT3 in cardiac function (1, 47), and the findings of Cabanelas et al. in their study provided some implications to the potential mechanism (48). Furthermore, the negative association between RDW and thyroid autoimmunity was also intriguing, which is the main cause of SHypo (49). The result may be ascribed to the following reasons. First, thyroid ultrasonographic scan indexes were unavailable in the NHANES database, which may lead to the underdiagnosed thyroid autoimmunity in our population (27). Moreover, the clinical conditions or laboratory anomalies that may result in elevated serum thyrotropin levels (49) were also lacked in the NHANES database.

The interpretation of this investigation should be in light of some shortcomings. First, this work was limited to the cross-sectional study design, thereby, we cannot establish the causal connections between RDW and thyroid function. Moreover, longitudinal follow-up data also lacked in this study. Second, the negative association between RDW and thyroid autoimmunity should be cautiously interpreted, and the studies with larger sample size for the specific disease are needed in the future. Finally, it is believed that RDW is a significant parameter with wide-ranging biological implications, which can be influenced by a variety of metabolic factors (1). Unfortunately, the potential confounding factors, such as erythrocyte sedimentation rate (ESR) (19), were not included in the present study.

## Conclusion

In conclusion, in this study by a nationally representative sample of the US general adult population, we confirmed a strong association between RDW and thyroid function, and then we demonstrated a J-shaped pattern between RDW and TSH concentrations. In addition, future studies are needed to elucidate the underlying mechanisms of the peculiar RDW-fT3 relationship. Moreover, we suggested thorough prospective investigations relating the role of RDW as a predictive marker of subclinical hypothyroidism because of the significant linkage the current study revealed.

## Data Availability Statement

The raw data supporting the conclusions of this article will be made available by the authors, without undue reservation.

## Author Contributions

All authors listed have made a substantial, direct, and intellectual contribution to the work and approved it for publication.

## Funding

This work was supported by grants from the Natural Science Foundation of Jiangsu Province (BK20181506).

## Conflict of Interest

The authors declare that the research was conducted in the absence of any commercial or financial relationships that could be construed as a potential conflict of interest.

## Publisher’s Note

All claims expressed in this article are solely those of the authors and do not necessarily represent those of their affiliated organizations, or those of the publisher, the editors and the reviewers. Any product that may be evaluated in this article, or claim that may be made by its manufacturer, is not guaranteed or endorsed by the publisher.
