# Supplementary material for: Association Between Red Blood Cell Distribution Width and Thyroid Function
Source: Front Endocrinol (Lausanne). 2022 Jan 18;12:807482. doi: 10.3389/fendo.2021.807482 (PMC8805204; doi:10.3389/fendo.2021.807482)
Supplement: Supplementary file 1 [file Table_1.docx]

Table S1 Baseline characteristics of NHANES (2007-2010) study population in RDW quartiles

| Characteristics | RDW quartiles | | | | | *p-Value* |
| --- | --- | --- | --- | --- | --- | --- |
|  | Overall | Q1 | Q2 | Q3 | Q4 |  |
| n | 6895 | 1593 | 1678 | 1879 | 1745 |  |
| RDW (%) | 12.6 (10.8-37.8) | 10.8-12.1 | 12.2-12.5 | 12.6-13.2 | 13.3-37.8 |  |
| **Demographics** | | | | | | |
| Age, years | 47.00 [32.00-63.00]^a^ | 38.00 [27.00-52.00] | 44.00 [31.00-60.00] | 51.00 [37.00-66.00] | 55.00 [39.00-70.00] | <0.001 |
| Gender (%) |  |  |  |  |  | <0.001 |
| Male | 3554 (51.5%)^b^ | 830 (52.1%) | 955 (56.9%) | 1000 (53.2%) | 769 (44.1%) |  |
| Female | 3341 (48.5%) | 763 (47.9%) | 723 (43.1%) | 879 (46.8%) | 976 (55.9%) |  |
| Ethnicity (%) |  |  |  |  |  | <0.001 |
| Non-Hispanic White | 3180 (46.1%) | 817 (51.3%) | 812 (48.4%) | 874 (46.5%) | 677 (38.8%) |  |
| Non-Hispanic Black | 1326 (19.2%) | 171 (10.7%) | 213 (12.7%) | 368 (19.6%) | 574 (32.9%) |  |
| Mexican American | 1264 (18.3%) | 322 (20.2%) | 357 (21.3%) | 344 (18.3%) | 241 (13.8%) |  |
| Other Hispanic | 794 (11.5%) | 196 (12.3%) | 198 (11.8%) | 212 (11.3%) | 188 (10.8%) |  |
| Other race | 331 (4.8%) | 87 (5.5%) | 98 (5.8%) | 81 (4.3%) | 65 (3.7%) |  |
| Education (%) |  |  |  |  |  | <0.001 |
| < High school diploma | 2141 (31.1%) | 414 (26.0%) | 493 (29.4%) | 607 (32.3%) | 627 (35.9%) |  |
| High school diploma | 1679 (24.4%) | 399 (25.0%) | 382 (22.8%) | 476 (25.3%) | 422 (24.2%) |  |
| > High school diploma | 3068 (44.5%) | 779 (48.9%) | 802 (47.8%) | 794 (42.3%) | 693 (39.7%) |  |
| Unknown | 7 (0.1%) | 1 (0.1%) | 1 (0.1%) | 2 (0.1%) | 3 (0.2%) |  |
| Marital status (%) |  |  |  |  |  | 0.065 |
| Married | 3486 (50.6%) | 815 (51.2%) | 871 (51.9%) | 953 (50.7%) | 847 (48.5%) |  |
| Unmarried | 3075 (44.6%) | 663 (41.6%) | 726 (43.3%) | 853 (45.4%) | 833 (47.7%) |  |
| Unknown | 334 (4.8%) | 115 (7.2%) | 81 (4.8%) | 73 (3.9%) | 65 (3.7%) |  |
| PIR (%) |  |  |  |  |  | 0.051 |
| 0-1 | 1399 (20.3%) | 287 (18.0%) | 326 (19.4%) | 393 (20.9%) | 393 (22.5%) |  |
| >1 | 4887 (70.9%) | 1169 (73.4%) | 1201 (71.6%) | 1322 (70.4%) | 1195 (68.5%) |  |
| Unknown | 609 (8.8%) | 137 (8.6%) | 151 (9.0%) | 164 (8.7%) | 157 (9.0%) |  |
| MAP, mmHg | 87.00 [80.00-94.00] | 85.00 [78.00-93.00] | 87.00 [80.00-94.00] | 87.00 [80.00-95.00] | 87.00 [80.00-96.00] | <0.001 |
| BMI, kg/m² | 27.70 [24.09-32.02] | 26.54 [23.40-30.28] | 27.56 [24.07-31.51] | 28.00 [24.17-32.22] | 29.12 [24.91-33.99] | <0.001 |
| Alcohol use (%) |  |  |  |  |  | <0.001 |
| Never or not in last 1 year | 2014 (29.2%) | 362 (22.7%) | 426 (25.4%) | 625 (35.8%) | 601 (32.0%) |  |
| 1 drink/d | 1312 (19.0%) | 301 (18.9%) | 300 (17.9%) | 371 (19.7%) | 340 (19.5%) |  |
| 2–4 drinks/d (men), 2–3 (women) | 1866 (27.1%) | 469 (29.4%) | 500 (29.8%) | 506 (26.9%) | 391 (22.4%) |  |
| >4 drinks/d (men), >3 (women) | 854 (12.4%) | 238 (14.9%) | 255 (15.2%) | 200 (10.6%) | 161 (9.2%) |  |
| Unknown | 849 (12.3%) | 223 (14.0%) | 197 (11.7%) | 201 (10.7%) | 228 (13.1%) |  |
| Smoke (%) |  |  |  |  |  | 0.019 |
| Never | 3429 (49.7%) | 830 (52.1%) | 849 (50.6%) | 942 (50.1%) | 808 (46.3%) |  |
| Former | 1709 (24.8%) | 358 (22.5%) | 395 (23.5%) | 458 (24.4%) | 498 (28.5%) |  |
| Current | 1499 (21.7%) | 324 (20.3%) | 368 (21.9%) | 419 (22.3%) | 388 (22.2%) |  |
| Unknown | 258 (3.7%) | 81 (5.1%) | 66 (3.9%) | 60 (3.2%) | 51 (2.9%) |  |
| Cancer (%) |  |  |  |  |  | <0.001 |
| Yes | 599 (8.7%) | 99 (6.2%) | 121 (7.2%) | 167 (8.9%) | 212 (12.2%) |  |
| No | 5950 (86.3%) | 1377 (86.4%) | 1474 (87.8%) | 1635 (87.0%) | 1464 (83.9%) |  |
| Unknown | 346 (5.0%) | 117 (7.4%) | 83 (5.0%) | 77 (4.1%) | 69 (3.9%) |  |
| Coronary heart disease (%) |  |  |  |  |  | <0.001 |
| Yes | 246 (3.6%) | 23 (1.4%) | 44 (2.6%) | 76 (4.1%) | 103 (5.9%) |  |
| No | 6288 (91.2%) | 1453 (91.2%) | 1547 (92.2%) | 1722 (91.6%) | 1566 (89.7%) |  |
| Unknown | 361 (5.2%) | 117 (7.4%) | 87 (5.2%) | 81 (4.3%) | 76 (4.4%) |  |
| Congestive heart failure (%) |  |  |  |  |  | <0.001 |
| Yes | 190 (2.8%) | 12 (0.8%) | 16 (1.0%) | 53(2.8%) | 109 (6.3%) |  |
| No | 6347 (92.0%) | 1465 (92.0%) | 1576 (93.9%) | 1746 (92.9%) | 1560 (89.4%) |  |
| Unknown | 358 (5.2%) | 116 (7.2%) | 86 (5.1%) | 80 (4.3%) | 76 (4.3%) |  |
| Angina pectoris (%) |  |  |  |  |  | <0.001 |
| Yes | 158 (2.3%) | 21 (1.3%) | 26 (1.6%) | 46 (2.5%) | 65 (3.7%) |  |
| No | 6385 (92.6%) | 1456 (91.4%) | 1567 (93.4%) | 1751 (93.2%) | 1611 (92.3%) |  |
| Unknown | 352 (5.1%) | 116 (7.3%) | 85 (5.0%) | 82 (4.3%) | 69 (4.0%) |  |
| **Biomarker** | | | | | | |
| Hemoglobin, g/dl | 14.30 [13.20-15.40] | 14.60 [13.70-15.60] | 14.70 [13.60-15.60] | 14.30 [13.40-15.30] | 13.40 [12.30-14.60] | <0.001 |
| MCV, fl | 89.00 [85.90-92.20] | 90.30 [87.70-92.90] | 89.40 [86.90-92.40] | 89.10 [86.40-92.00] | 86.30 [81.90-90.70] | <0.001 |
| Total cholesterol, mg/dl | 191.00 [165.00-221.00] | 190.00 [164.00-218.00] | 193.00 [167.00-222.00] | 194.00 [167.00-223.00] | 189.00 [161.00-220.00] | 0.002 |
| HDL-C, mg/dl | 50.00 [41.00-61.00] | 49.00 [40.00-59.00] | 49.00 [41.00-59.00] | 50.00 [41.00-62.00] | 50.00 [41.00-62.00] | 0.014 |
| Triglycerides, mg/dl | 124.00 [81.00-192.00] | 125.00 [79.00-197.25] | 127.00 [83.00-199.00] | 123.00 [82.00-194.00] | 121.00 [81.00-179.00] | 0.09 |
| Serum albumin, g/l | 4.30 [4.00-4.50] | 4.30 [4.10-4.50] | 4.30 [4.10-4.50] | 4.20 [4.00-4.40] | 4.10 [3.90-4.30] | <0.001 |
| BUN, mg/dl | 12.00 [9.00-15.00] | 11.00 [9.00-14.00] | 12.00 [10.00-15.00] | 12.00 [10.00-16.00] | 12.00 [9.00-16.00] | <0.001 |
| Serum iron, ug/dl | 81.00 [61.00-105.00] | 91.00 [71.00-115.00] | 88.00 [68.00-113.00] | 80.00 [63.00-102.00] | 66.00 [46.00-89.50] | <0.001 |
| Folate, nmol/l | 1060.00 [795.00-1420.00] | 1055.00 [812.00-1400.00] | 1060.00 [805.00-1400.00] | 1050.00 [793.00-1410.00] | 1060.00 [773.75-1460.00] | 0.876 |
| Serum cotinine, ng/ml | 0.06 [0.02-18.40] | 0.06 [0.02-10.10] | 0.06 [0.02-19.65] | 0.06 [0.02-21.30] | 0.07 [0.02-48.08] | 0.012 |
| Estimated GFR, ml s^−1^m^−2^ | 1.59 [1.32-1.88] | 1.67 [1.43-1.95] | 1.64 [1.36-1.91] | 1.55 [1.29-1.85] | 1.51 [1.20-1.84] | <0.001 |
| CRP, mg/dl | 0.18 [0.07-0.44] | 0.13 [0.05-0.30] | 0.16 [0.06-0.37] | 0.20 [0.08-0.43] | 0.28 [0.11-0.69] | <0.001 |
| Urine iodin, ug/l |  |  |  |  |  | <0.001 |
| < 99 | 2025 (29.4%) | 525 (33.0%) | 489 (29.1%) | 507 (27.0%) | 504 (28.9%) |  |
| 99-199 | 2216 (32.1%) | 524 (32.9%) | 534 (31.8%) | 612 (32.6%) | 546 (31.3%) |  |
| >199 | 2389 (34.6%) | 494 (31.0%) | 598 (35.6%) | 693 (36.9%) | 604 (34.6%) |  |
| Unknown | 265 (3.8%) | 50 (3.1%) | 57 (3.4%) | 67 (3.6%) | 91 (5.2%) |  |

^a^ Weighted median [95% CI for median].

^b^ Actual frequency (weighted percentage).

Abbreviations: NHANES, National Health and Nutrition Examination Survey; RDW, red blood cell distribution width; PIR, poverty-to-income ratio; MAP, mean arterial pressure; BMI, body mass index; MCV, mean corpusular volume; HDL-C, high-density lipoprotein cholesterol; BUN, blood urea nitrogen; CRP, C-reactive protein; estimated GFR, estimated glomerular filtration rate.
